# Urinary Tract Infection in Neurologic Patients Undergoing Intermittent Catheterization: A Systematic Review and Meta-Analysis of Catheter Type and Technique

**DOI:** 10.7759/cureus.101064

**Published:** 2026-01-08

**Authors:** Sara Skalli, Ihssane Hmamouchi, Redouane Abouqal, Najia Hajjaj-Hassouni, Samia Karkouri

**Affiliations:** 1 Physical and Rehabilitation Medicine, Ibn Sina University Hospital, Faculty of Medicine and Pharmacy of Rabat, Mohammed V University, Rabat, MAR; 2 Clinical Epidemiology, Faculty of Medicine, Health Sciences Research Center, International University of Rabat (UIR), Rabat, MAR; 3 Laboratory of Biostatistics, Clinical, and Epidemiological Research, Ibn Sina University Hospital, Faculty of Medicine and Pharmacy of Rabat, Mohammed V University, Rabat, MAR; 4 Faculty of Medicine, Health Sciences Research Center, International University of Rabat (UIR), Rabat, MAR

**Keywords:** hydrophilic catheter, intermittent catheterization, neurogenic bladder, spinal cord injury, urinary tract infection

## Abstract

Intermittent urinary catheterization (IUC) is one of the recommended methods for bladder emptying in patients with bladder disorders. To our knowledge, no meta-analysis has yet evaluated the risk of urinary tract infections (UTIs) in adult neurological patients according to the type of catheter and IUC method.

To address this knowledge gap, a systematic review of the literature was conducted using the PubMed, Embase, and Cochrane Trials CENTRAL databases, according to Preferred Reporting Items for Systematic Reviews and Meta-Analyses (PRISMA) recommendations. We included randomized clinical trials and cohort studies comparing at least two types of catheters and/or two IUC strategies in adult patients. Only studies published in French or English were considered. Heterogeneity was assessed using the I² statistic. A random-effects model was applied to estimate the pooled effect size across studies. Publication bias was assessed using the Luis Furuya-Kanamori (LFK) index and the Doi plot. All statistical analyses were performed using R software, version 4.3.2 (The R Foundation for Statistical Computing, Vienna, Austria). The study was registered with PROSPERO (CRD42023481012).

Nine studies were included in the systematic review, of which seven (six randomized controlled trials and one cohort study) were eligible for meta-analysis. The estimated pooled odds ratio based on the random-effects model was 0.53 (95% CI: 0.34-0.84), with moderate heterogeneity (I² = 49%). Publication bias was substantial.

These findings suggest that hydrophilic catheters are associated with a lower risk of symptomatic UTI compared with uncoated ones. Further research is needed to evaluate reusable catheter strategies, considering economic and environmental factors.

## Introduction and background

Intermittent urinary catheterization (IUC) is recommended for managing bladder disorders, especially neurological ones [1]. This technique involves briefly inserting a catheter at regular intervals to completely empty the bladder [2]. It ensures clean and complete urination, reducing infections and improving quality of life [3]. Performing IUC requires physical, cognitive, and functional capacities. Self-catheterization occurs when the individual independently performs the procedure, while, in the case of significant impairment, a third party may perform it, known as hetero-catheterization. There are two main types of catheters [4]: uncoated (with or without pre-lubricated systems) and coated (hydrophilic or non-hydrophilic). They can be made from clear plastic polyvinyl chloride (PVC) or PVC-free materials.

IUC can be conducted using three techniques. Firstly, the sterile technique involves sterile gloves, a sterile container, and perineal disinfection with an antiseptic solution. Secondly, the aseptic technique requires a sterile single-use catheter, perineal disinfection with an antiseptic solution, and a no-touch technique avoiding direct manual contact with the catheter. Lastly, the clean technique involves clean hands and a clean container, along with perineal cleaning without an antiseptic solution. Catheters may be used once or reused for multiple subsequent catheterizations. Reused catheters can be cleaned with soap and water, soaked in a disinfectant solution, boiled, heated in a microwave, and stored for the next use [5]. Practices vary around the world, and there is no clear consensus.

Urinary tract infections (UTIs) pose a significant concern for IUC users, especially neurological patients with spinal cord injury (SCI) [6]. The Infectious Diseases Society of America (IDSA) defines catheter-associated UTI as the presence of symptoms or signs consistent with a urinary infection, with no identified source of infection, along with ≥10³ colony-forming units (CFU)/mL of ≥1 bacterial species in urine samples obtained from a catheter [7]. Symptoms may include fever, altered mental status, unexplained fatigue or lethargy, flank pain, acute hematuria, pelvic discomfort, dysuria, urgency, or frequent, painful, or sensitive suprapubic pain, especially in individuals who have had their catheter removed. For persons with SCI, the UTI definition also considers worsening spasticity, autonomic dysreflexia (sweating, bradycardia, increased blood pressure), or general malaise.

Despite scientific advances in catheter design and materials, there is no unanimous consensus regarding IUC practices. This systematic review and meta-analysis aimed to examine data regarding UTIs in adult neurogenic patients undergoing IUC, based on catheter type and catheterization technique.

## Review

Methods

Review Design and Registration

This systematic review and meta-analysis were conducted according to the Preferred Reporting Items for Systematic Reviews and Meta-Analyses (PRISMA) guidelines [8]. The review protocol was registered with PROSPERO under registration number CRD42023481012.

Search Strategy

A systematic literature search was performed in January 2023 using PubMed, the Excerpta Medica database (Embase), and the Cochrane CENTRAL trials. The search formula incorporated keywords combined with Medical Subject Headings (MeSH) terms: IUC, UTI, and neurogenic urinary disorders (Table 1).

**Table 1:** Search strategy

	Terms
1	(intermittent catheterization) OR (intermittent urinary catheterization)
2	(urinary tract infection) OR (urinary infection) OR (symptomatic urinary infection)
Population (P)	Neurologic adult patients with neurogenic urinary disorders
Intervention (I)	Intermittent catheterization
Comparison (C)	Type of catheter and/or technique of catheterization
Outcomes (O)	Urinary tract infection

Study Selection

Randomized clinical trials, as well as retrospective and prospective cohort studies, were included if they compared at least two types of catheters and/or two techniques of IUC in adult patients with neurogenic bladder. Participants included adults requiring IUC for neurogenic bladder management.

Selection Criteria

Inclusion criteria were articles published after 2000, up to January 2023, available in full text in French or English, involving neurogenic bladder disorders requiring IUC, and comparing at least two types of catheters and/or two techniques of IUC.

Exclusion criteria included letters to the editor, case reports, posters, recommendations from societies and experts, systematic reviews and meta-analyses, studies involving antiseptic or antibiotic prophylaxis, and studies on the pediatric population.

Outcome

The outcome analyzed was symptomatic UTI. Other outcomes, such as hematuria, patient satisfaction, preferences, quality of life, and economic outcomes, were not analyzed.

Data Extraction

Data collection followed PRISMA recommendations. Two authors (SS and SK) independently identified eligible papers based on title and abstract, and any disagreements were resolved by a third author (IH). Extracted data were entered into a pre-set table, and the manuscript was written according to PRISMA guidelines [8].

Risk of Bias Assessment

For randomized controlled trials, the Revised Cochrane Risk of Bias tool for randomized controlled trials (RoB 2) was employed [9]. For cohort studies, the Newcastle-Ottawa Scale was used [10].

Statistical Analysis

The analysis focused on research methodology, study population, catheter type, technique, UTI definition, and authors' conclusions. The odds ratio was the outcome measure. Statistical heterogeneity among included studies was estimated using I² (I² > 50% indicates significant heterogeneity). Due to expected heterogeneity, a random-effects model was adopted. Doi plots were used to assess publication bias based on the number of studies included in the meta-analysis, with Luis Furuya-Kanamori (LFK) index values outside the range of -1 to +1 indicating asymmetry and, therefore, significant publication bias [11]. Statistical analyses were performed using R software, version 4.3.2 (The R Foundation for Statistical Computing, Vienna, Austria) [12].

Results

Study Selection

**Figure 1:**
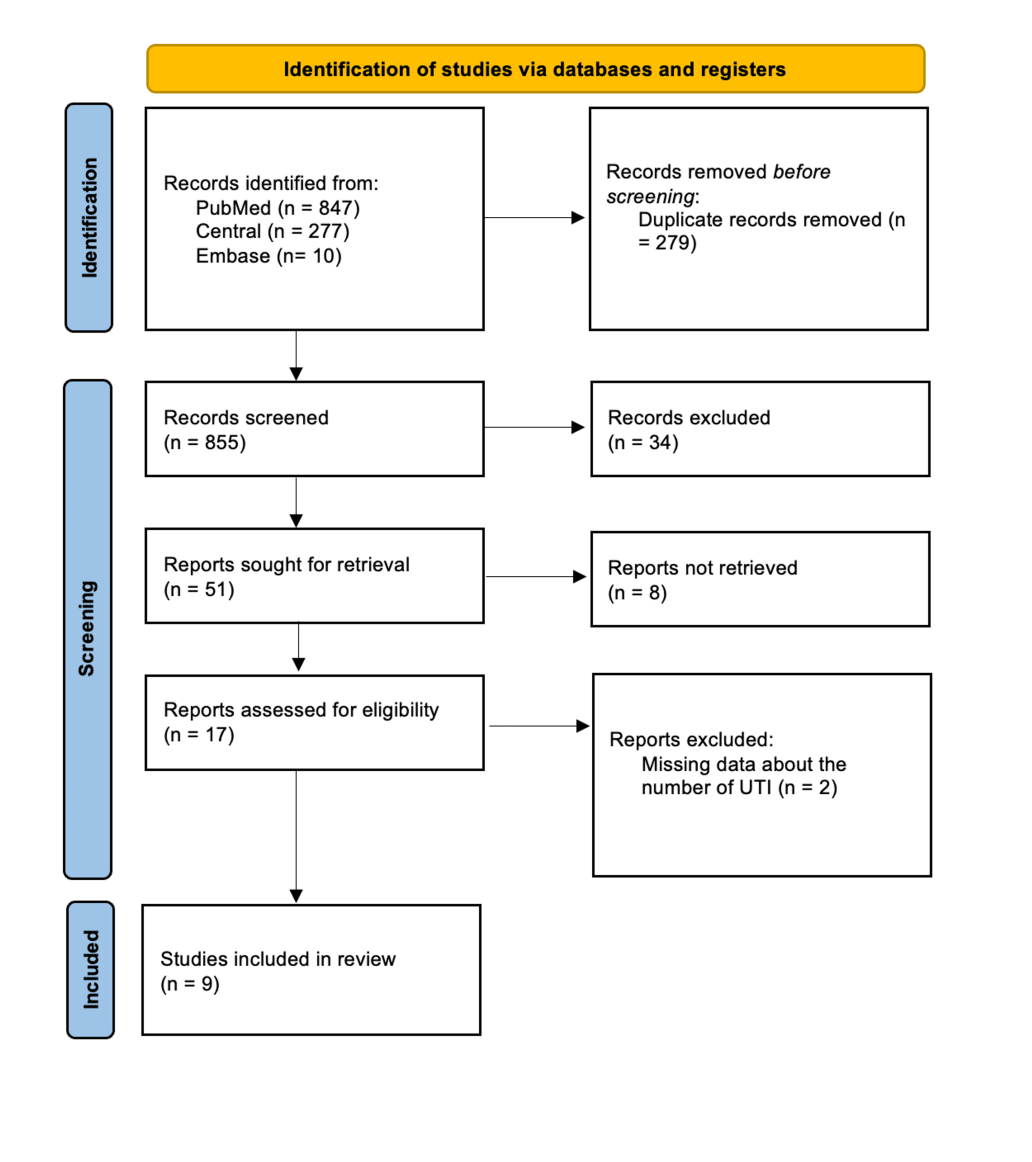
PRISMA flow diagram

The electronic searches identified 1,134 articles, resulting in 855 articles after duplicate removal. Subsequently, 804 articles were screened through title and abstract review based on predefined criteria. Of these, 17 of the 51 studies were eligible for full-text screening, and nine studies were ultimately included in the narrative review. The reasons for exclusion are detailed in Figure 1.

For the meta-analysis, seven studies were included, while Spinu et al. (2012) [13] was excluded due to missing UTI data in each group, and Moore et al. (2006) [14] was excluded as it focused solely on the IUC technique. The flow of literature through the review process is illustrated in Figure 1 [8,15].

Study Characteristics

Sample sizes varied, with a median of 50 (36-123). Seven studies involved patients with SCI. Chartier-Kastler et al. (2022) [16] included people with neurogenic bladder without a specified etiology, and in Vapnek et al. (2003) [17], 56 individuals had SCI, while five had an unspecified neurogenic disorder. Age ranged from 22 to 75 years, with gender distribution varying across studies, predominantly including men. There was diversity in the duration of disease and IUC, as summarized in Appendices 1 and 2.

Risk of Bias Assessment

Randomized controlled trials were evaluated using the RoB2 tool, and cohort studies were evaluated using the Newcastle-Ottawa Scale. Consensus findings are presented in Tables 2-3. Most of the included studies used appropriate randomization techniques, but biases in detection and performance were more common. These results suggest that outcomes should be interpreted cautiously. Regarding the cohort studies, they exhibited overall moderate methodological quality, as both studies indicated sufficient representativeness of the exposed group and appropriate measurement of exposure. Nonetheless, constraints concerning the comparability of cohorts decreased confidence in the strength of their results.

**Table 2:** RoB2 for assessing randomized controlled trials

Author	Study type	Random sequence generation (selection bias)	Allocation concealment (selection bias)	Blinding of participants and personnel (performance bias)	Blinding of outcome assessment (detection bias = self-reported)	Incomplete outcome data (attrition bias)	Selective reporting (reporting bias)	Other bias
Giannantoni et al. (2001) [18]	Randomized crossover study	Low risk	Low risk	Low risk	Low risk	Low risk	Low risk	Some concerns
Vapnek et al. (2003) [17]	RCT	Some concerns	Low risk	Some concerns	Low risk	High risk	Low risk	Low risk
De Ridder et al. (2005) [19]	RCT	Low risk	Low risk	Some concerns	Low risk	High risk	Low risk	Low risk
Moore et al. (2006) [14]	RCT	Low risk	Low risk	Some concerns	Low risk	Low risk	Low risk	Low risk
Cardenas and Hoffman (2009) [20]	RCT	Low risk	Some concerns	High risk	High risk	Low risk	Low risk	Low risk
Sarica et al. (2010) [21]	RCT	Low risk	Some concerns	High risk	Some concerns	High risk	Low risk	Low risk
Cardenas et al. (2011) [22]	RCT	Low risk	Low risk	High risk	Some concerns	High risk	Low risk	Low risk

**Table 3:** Newcastle-Ottawa for assessing cohort studies

Study	Study design	Selection				Comparability	Outcome			
		Representativeness of the exposed cohort	Selection of the non-exposed cohort	Ascertainment of exposure	Demonstration that the outcome of interest was not present at the start of the study	Comparability of cohorts on the basis of the design or analysis	Assessment of outcome	Was the follow-up long enough for outcomes to occur	Adequacy of follow-up of cohorts	Quality score
Spinu et al. (2012) [13]	Cohort study	1	-	-	-	2	-	1	-	4
Chartier-Kastler et al. (2022) [16]	Cohort study	1	1	1	1	2	1	1	1	7

Meta-Analysis on UTI and Type of Catheter

Given the limited number of studies comparing catheter insertion techniques, the meta-analysis focused exclusively on catheter types. A total of k = 7 studies were included in the analysis, involving a total sample size of 1,043. Spinu et al. (2012) [13] and Moore et al. (2006) [14] were excluded due to missing data and/or an exclusive focus on the IUC technique, respectively. Due to the limited number of studies, subgroup analysis was not possible.

**Figure 2:**
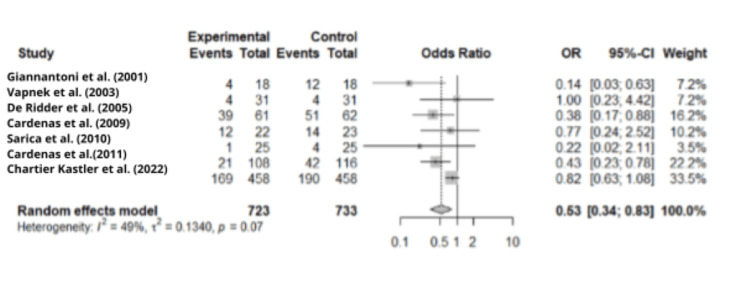
Forest plot of urinary tract infection according to type of catheter

**Figure 3:**
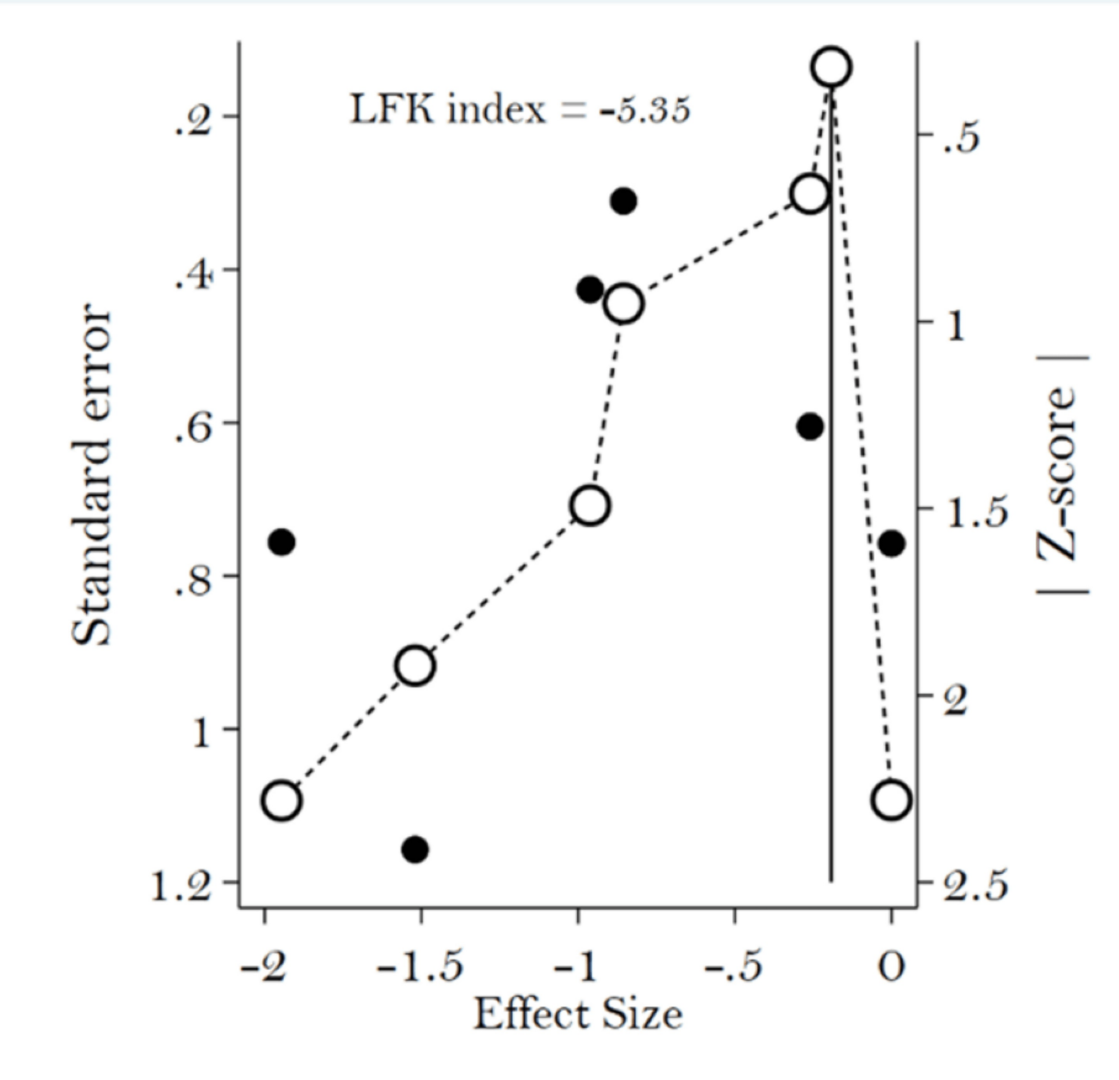
Bias of publication assessed with Doi plot LFK index, Luis Furuya-Kanamori Index

The odds ratio was 0.53 (95% CI: 0.34-0.83). Analysis of the forest plot revealed heterogeneity among the included studies, with I² = 49%, approaching the threshold of significance (Figure 2). Publication bias, evaluated by the LFK index, was greater than |2|, as shown in the Doi plot (Figure 3).

Discussion

This meta-analysis found that the use of hydrophilic catheters reduces UTI, but the sample size remained limited, studies were at moderate risk of bias, and publication bias was significant.

In terms of the methodology in the selected studies, seven of them were randomized clinical trials [14,17-22], while two were retrospective studies [13,16].

There is heterogeneity in patient characteristics, including age, gender distribution, disease duration, and IUC. This diversity may affect the generalizability of the results and should be considered when interpreting findings. Among the study population, seven studies involved patients with SCI. This focus on SCI reflects the high-risk profile of urinary and renal complications, highlighting the importance of early intervention. Indeed, research was predominantly conducted in hospital and short-term outpatient settings, except for the studies conducted by Spinu et al. (2012) [13] and Chartier-Kastler et al. (2022) [16], which focused on short- to medium-term IUC (within one year).

Regarding comparison based on catheter type: in the comparison of coated non-hydrophilic and uncoated catheters [18], the non-hydrophilic catheter showed superiority, albeit with a small sample size. As for the comparison between hydrophilic and coated non-hydrophilic catheters in Sarica et al. (2010) [21] and Chartier-Kastler et al. (2022) [16], no significant difference was found, although the pre-lubricated catheter demonstrated fewer UTIs. Spinu et al. (2012) [13] reported minimal differences. Finally, coated hydrophilic catheters were superior to uncoated ones in studies by Vapnek et al. (2003) [17], De Ridder et al. (2005) [19], and Cardenas et al. (2011) [22], with Cardenas and Hoffman (2009) [20] revealing no difference in symptomatic UTI numbers. However, the treatment of UTI with antibiotics was significantly reduced in the hydrophilic group.

Regarding IUC techniques, single-use no-touch was found superior to the sterile technique by Giannantoni et al. (2001) [18]. Vapnek et al. (2003) [17] reported no difference in urinary infection rates between single-use and multiple-use catheters, but the reduction was more significant in the single-use (hydrophilic) group. In contrast to hydrophilic catheters, dry catheters can be used multiple times. Moore et al. (2006) [14] revealed that the single-use clean technique resulted in fewer urinary infections compared to the sterile technique.

Several factors are considered when determining the reliability of meta-analysis results. First is the nature of the research. We included clinical trials with varying risk of bias, from low to moderate. The included cohort was of good quality. Second, the effects measured by odds ratios were accurate and had correct confidence intervals. Third, the endpoint definition of symptomatic UTI was clinically and biologically consistent across all studies included in the meta-analysis. However, it is important to note that there was a significant risk of publication bias. Therefore, our meta-analysis shows moderate confidence in the superiority of hydrophilic catheters in reducing UTIs in neurological patients undergoing intermittent catheter therapy.

Regarding catheter insertion technique, results did not support the sterile technique, and multiple use of catheters did not lead to an increase in UTIs. Current clinical trials, including MultiCath ISRCTN42028483 [23] in 520 patients and COMPARE NL8296 [24] in 456 patients, are aiming to evaluate multiple-use versus single-use catheters, with symptomatic UTI as the primary outcome. Notably, cost considerations in two studies indicated that clean catheters were less expensive than sterile ones [14], while hydrophilic and lubricated catheters were more expensive than dry ones [21]. From an ecological and economic perspective, future studies should focus on comparing multiple-use versus single-use catheters, especially in countries where catheters are not reimbursed, and the population has a low economic status.

## Conclusions

IUC stands as a recommended technique for ensuring complete and efficient bladder emptying in neurological conditions. Various materials and techniques can be employed. Hydrophilic catheters appear to be associated with a lower risk of symptomatic UTI compared with uncoated ones, although the evidence is limited by a moderate risk of bias and potential publication bias. However, considering economic and environmental issues, forthcoming studies should prioritize the comparison of multiple-use versus single-use clean techniques, ensuring a substantial sample size and a unified definition of UTI.

## Appendices

Appendix 1

**Table 4:** Patients characteristics of studies included SCI, Spinal Cord Injury; IC, Intermittent Catheterization

Author	Setting	Disease	Total, N	Women, N	Men, N	Age	Disease duration	Intermittent catheterization duration
Giannantoni et al. (2001) [18]	Hospital	SCI recently injured	18	2	16	38.2 ± 16.4	37.4 days, SD 13.6	-
Vapnek et al. (2003) [17]	Outpatient clinic at 3 institutions	Neurogenic bladder (56 SCI), 5 other neurogenic disorders not specified)	62	0	62	Interv: 39.9 ± 12.9; Comp: 39.6 ± 16	-	Interven 43.7(1_161); Compar 56(4_228) range
De Ridder et al. (2005) [19]	Hospital than discharged (ambulatoire)	SCI injured less than 6 months	123	0	123	Intervent: 37.5 ± 14.6; Comp: 36.7 ±14.6	Less than 6 months	-
Moore et al. (2006) [14]	Hospital rehabilitation units	Cervical SCI recently injured	36	8	28	40 ± 16.7	6.6 weeks, SD 4	-
Cardenas and Hoffman (2009) [20]	Community	SCI	56	-	-	Interv 42.3±10.4; Compar 40.1 ±14.8	Inter 15.2, SD 10.5; Compar 16.1, SD 14.7	-
Sarica et al. (2010) [21]	Hospital and inpatient clinic	SCI	25			37.04 ± 11.86		
Cardenas et al. (2011) [22]	Institutional and community multicenter	SCI	224	-	-	Inter 37.2 ± 14.4; compar 35.1 ± 13.2	Interv 32 days (20_47); Comp 29 (16_52)	Comp 5 days (2_7); Interent 5 (2_8)
Spinu et al. (2012) [13]	-	SCI	45	-	-	Inter 45 ± 13.6; Comp 47 ± 13.1	Long term	-
Chartier-Kastler et al. (2022) [16]	-	Neurological dysfunction leading to IC	916	541	375	58 ± 17	At least 1 year	-

Appendix 2

**Table 5:** Methodology of included studies SCI, Spinal Cord Injury; IC, Intermittent Catheterization; UTI, Urinary Tract Infection; PVC, Polyvinyl Chloride; CFU, Colony-Forming Units; FW, Follow-Up; RBC, Red Blood Cells; WBC, White Blood Cells; HPF, High-Power Field; ATC, Anatomical Therapeutic Chemical code; ICD-10, International Classification of Diseases, 10th Revision

Author	Population	Intervention	Comparison	UTI definition	UTI intervention group, N	UTI comparison group 1, N	p-value	Methods	Other outcomes	Inclusion criteria	Exclusion criteria	Conclusion
Giannantoni et al. (2001) [18]	SCI recently injured	(n = 18) coated prelubricated non-hydrophilic catheter, no-touch technique, single use	(n = 18) uncoated polyvinyl chloride (PVC) catheter, single use	Odorous urine, onset of urinary incontinence, increased spasticity, autonomic dysreflexia, increased sweating, malaise, pyuria, significant bacteruria	4 (7.4%)	12 (22.2%)	0.03	Initial course was randomized; 7 weeks for each catheter; KT every 5 hours. FW 2, 4, and 7 weeks for each type, so 2, 4, 7, 9, 11, 14 weeks	Urethral cell count, satisfaction	-	-	Coated prelubrificated non-hydrophilic > coated PVC
Vapnek et al. (2003) [17]	56 SCI, 5 other neurogenic disorders not specified	(n = 31) hydrophilic coated plastic clean technique single use	(n = 31) polyvinyl chloride catheter uncoated multiple use (4 to 5 times)	Bacterial colony count of 100,000 cfu or greater and at least 1 clinical symptom, such as fever, chills, malodorous urine, increased spasticity, or malaise	4.03	4.34	0.012 for the intervention group vs p = 0.24 comparison group	Baseline: UTI based on self-reporting, ECBU FW every 3 months for 1 year, nb UTI/mois de KT, study the decrease of UTI rate in each group	Hematuria, pyuria, adverse event	Self-catheterization, male, neurogenic bladder	History of vesicourethral reflux, bladder calculi, unexplained hematuria, prophylactic antibiotics, and incapable of following	Hydrophilic coated clean technique single use > uncoated PVC catheter multiple use (4 to 5 times)
De Ridder et al. (2005) [19]	SCI injured less than 6 months ago	(n = 61) Hydrophilic-coated made with polyurethane and a hydrophilic coating made by polyvinyl pyrrolidone single use	(n = 62) uncoated polyvinyl chloride catheter manually lubricated (water-soluble gel)	A clinical infection with symptoms of UTI for which treatment was prescribed	39 (64%)	51 (82%)	0.02	-	Bleeding episodes, satisfaction, strictures, and the convenience of use	Male >16 years old SCI less than 6 months with neurogenic bladder that needs IC at least 3 times a day	Symptomatic UTI, prophylactic antiseptic or antibiotic treatment, permanent catheter for more than 10 days	Hyrophilic-coated > uncoated PVC catheter
Moore et al. (2006) [14]	Cervical SCI was recently injured	(n = 16) single-use clean technique with PVC catheter	(n = 20) single-use sterile technique PVC catheter	≥10^5^ (CFU)/mL, >10 leukocytes per HPF (pyuria), fever (> 38°C), general malaise, increased spasticity, and/or autonomic dysreflexia characterized by headache, flushing of the face and neck, sweating, an increase in blood pressure, and the presence of usual pathogens.	6 (16.67%)	9 (25%)	-	Standardized technique, every 4 to 6 hours, weekly urine specimens, 1-year follow-up (n = 18, 50%)	Time to onset UTI, bacteriuria, adverse effect, cost	Previously healthy adults with stated preinjury normal bladder function and no history of urinary tract infections, required intermittent catheterization by nursing staff every 4-6 hours, speak and read English	Prophylactic antibiotics, self-catheterizing or had a caregiver catheterizing them, developed a symptomatic urinary tract infection, were discharged from the hospital, or requested withdrawal	Clean > sterile
Cardenas and Hoffman (2009) [20]	SCI	(n = 22) hydrophilic catheter	(n = 23) uncoated catheter clean technique single use	Significant bacteriuria (105 CFU/mL) plus at least 1 sign or symptom suggestive of UTI	12 (54%)	14(61%)	0.67	At least 1 episode of UTI, FW 1, 2, 3, 6, 9, 12 months	Symptom qestionnaire	>18 years old SCI 6 months or more ago, self-reported history of 2 or more UTIs during the past year, use of IC with a noncoated catheter and an open system, no plan to change the method of bladder drainage during the study period, naive to hydrophilic catheters	Upper urinary tract abnormalities, renal or bladder calculi	No difference in the number of symptomatic UTIs in the 2 groups; it appears that the mean number of UTIs treated with antibiotics was significantly smaller in the hydrophilic group
Sarica et al. (2010) [21]	SCI	Hydrophilic-coated catheter single-use clean technique	Gel-lubricated non-hydrophilic catheter, single-use clean technique	Bacterial colony count of 100,000 cfu or greater and at least 1 clinical symptom, such as fever, chills, malodorous urine, increased spasticity, or malaise	1	4	>0.05	3 different types of catheters, order randomly, each catheter for 6 weeks, FW 6, 12, and 18 weeks	Urethral cytology, urinalysis (red blood cells, white blood cells, pyuria), patient, coast	Male > 18 years old, SCI less than 6 months, self-catheterization 4 to 6 times/day	Clinical urinary tract infection, unexplained hematuria, bladder calculi, urethral stenosis or fibrosis, mentally unstable patient, participation in another clinical trial, prophylactic antiseptic or antibiotic treatment, permanent catheter for more than 10 days	No difference between
Cardenas et al. (2011) [22]	SCI	(n = 108) hydrophilic coated catheter sterile single use polyurethane, coating: polyvinyl pyrrolidone	(n = 116) non-coated catheter sterile, single-use PVC	Clinical definition of symptomatic UTI: a) Antibiotic treatment has been prescribed. 2) Strict definition of symptomatic UTI: a) Antibiotic treatment has been prescribed; b) Bacteriuria 102 CFU/mL (in IC specimens); c) At least 1 of 7 UTI symptoms based on consensus guidelines (fever, autonomic dysreflexia [sweating, bradycardia, blood pressure elevation], increased spasticity, discomfort or pain over the kidney or bladder or during micturition, onset and/or increase in incontinence episodes, cloudy urine with increased odor, malaise, lethargy, or sense of unease); d) Dipstick test positive for leukocyte esterase	21	42	0.22 in the institutional period vs p = 0.248 full study period	Study period: 6 months, time to onset of first symptomatic UTI, incidence of UTI with a ratio of UTI/months	Microhematuria, satisfaction, safety outcomes = adverse events	SCI less than 3 months before inclusion, neurogenic bladder dysfunction due to the SCI, and IC required at least 3 times a day	Symptoms of UTI, treatment with prophylactic antibiotics, history of unresolved vesico-ureteral reflux and/or urolithiasis, IC for more than 10 days before study inclusion, pregnancy, or plans to become pregnant during the study period	Hydrophilic coated > PVC uncoated
Spinu et al. (2012) [13]	SCI	(n = 30) hydrophilic catheter	(n = 15) non-hydrophilic catheter	Urinary questionnaire	-	-	0.6274	Number of UTIs	Number of inflammatory episodes at the scortal level/year, number of bleeding episodes, preference, and satisfaction	-	-	Hydrophilic > non-hydrophilic (a slightly lower number of UTI)
Chartier-Kastler et al. (2022) [16]	Neurological dysfunction leading to IC	(n = 458) prelubricated catheter	(n = 458) hydrophilic catheter	(1) The prescription of an antibiotic whose sole indication is a UTI (based on the European guidelines) or (2) the prescription of a nonspecific antibiotic (ATC code) with a diagnosis (ICD-10 code) related to a UTI. If two ssUTIs were detected in the same patient within a 4-week period, those were considered to be a single event	169	190	p = 0.015	12 months pre-indexed period, 28 months inclusion, 12 months follow-up, extended 4 months follow-up. Analyze the study group as a whole, and the sub-population	Number of UTIs per patient, duration of use, daily frequency, switch rate	> 18 yr, at least one prescription of a prelubricated or hydrophilic catheter during the inclusion period, a preindex period of at least 1 yr, and data available for the main and extended follow-up periods	The prescription of two or more different types of catheters at the index date, no follow-up data during the follow-up period, and the extended follow-up period.	No difference for patients who experienced at least one UTI during 12 months follow-up, however, considering the continuous use subpopulation, patients with at least 1 UTI were lower in the prelubrificated catheter
